# D-Dimer Concentrations and Thromboelastography in Five Dogs With Ischemic Stroke

**DOI:** 10.3389/fvets.2019.00255

**Published:** 2019-08-06

**Authors:** Bodil Cathrine Koch, Luca Motta, Bo Wiinberg, Ulrik Westrup, Annemarie Thuri Kristensen, Geoff Skerritt, Mette Berendt, Hanne Gredal

**Affiliations:** ^1^Department of Veterinary Clinical Sciences, Faculty of Health and Medical Sciences, University of Copenhagen, University Hospital for Companion Animals, Frederiksberg, Denmark; ^2^ChesterGates Veterinary Specialists, Chester, United Kingdom; ^3^Novo Nordisk A/S, Måløv, Denmark

**Keywords:** ischemic stroke, infarction, cerebrovascular accident, hemostatic parameters, D-dimer, hypercoagulability, thromboelastography, TEG

## Abstract

Ischemic stroke is a condition increasingly recognized in dogs; however, the number of publications on dogs with ischemic stroke is still limited and hemostatic parameters are infrequently reported. D-dimer levels have been shown to be elevated in people with acute ischemic stroke compared to a healthy control population and it has been proposed that a normal D-dimer can be used to exclude thromboembolism in dogs. In this case series, we report hemostatic parameters, including D-dimer and thromboelastography (TEG) along with clinical and imaging findings for five dogs diagnosed with ischemic stroke. All dogs had a normal D-dimer concentration on presentation. A hypercoagulable state was identified in two dogs based on the results of the TEG, and was suspected in the remaining three cases based on a shortened TEG clot reaction time. Based on the findings in the present cases, a D-dimer within the normal reference range does not seem an appropriate negative predictor for canine ischemic stroke. The demonstration of a possible hypercoagulable state, as identified by the TEG, is an interesting finding which should be explored further to help reveal predisposing hypercoagulable conditions in dogs with ischemic stroke.

## Background

Ischemic stroke is defined as an acute interruption of the blood supply to a dependent area of the brain caused by a thrombotic or thromboembolic event, resulting in infarction with loss of neurological function of the related vascular territory (1). The condition is increasingly recognized in dogs, and the diagnosis is based on an acute onset of non-progressive neurological signs in combination with characteristic magnetic resonance imaging (MRI) findings (2, 3).

In humans, a reliable and inexpensive diagnostic test and predictor of prognosis and risk of recurrence is being sought for ischemic stroke (4). It has been shown that plasma D-dimer levels are elevated in acute ischemic stroke compared to healthy control populations (4–11). As D-dimers are solely derived from the degradation of cross-linked fibrin monomers in stable clots, they are specific for active coagulation and fibrinolysis. A normal D-dimer in humans is considered a valuable negative predictor for intravascular thrombotic processes, especially in cases if central venous thrombosis (12). However, normal D-dimer levels cannot rule out a diagnosis of ischemic stroke in people and should not replace clinical and radiological evaluation (4). The use of D-dimer has shown promising results when screening for thromboembolic disease and disseminated intravascular coagulation (DIC) in dogs, and it has been proposed that a normal D-dimer concentration can be used to exclude thromboembolism in suspected cases (13, 14).

Thromboelastography (TEG) is a hemostatic diagnostic tool used for assessment of coagulation. It is an analytical method that enables global assessment of hemostatic function with evaluation of both plasma and cellular components (15). TEG has been successfully validated for the assessment of DIC and bleeding disorders in dogs (16, 17), and the assay has gained recent practical application in people with acute ischemic stroke for assessing coagulation status and response to thrombolytic therapy (18).

The number of publications in dogs with ischemic stroke where hemostatic parameters have been reported is limited (19–21). The usefulness of coagulation tests such as D-dimer concentrations and TEG in the diagnosis and management of canine ischemic stroke is therefore still uncertain. In this case series we describe clinical and MRI findings as well as hemostatic parameters including D-dimer and TEG for five dogs diagnosed with ischemic stroke.

## Case Presentations

For the following cases TEG measurements were compared with a previously established reference range for citrated plasma (22) and whole blood (23). TEG was performed on citrated plasma in cases 1–3, as the samples needed to be frozen for transport before analysis could take place, and on citrated whole blood in cases 4–5.

### Case 1

A 13-year, nine-month-old male neutered Yorkshire terrier presented with a peracute onset of left-sided circling, and a history of polyuria and polydipsia. The general physical examination identified a grade III/VI systolic heart murmur and obesity. On neurological examination, the dog was obtunded and was circling in small circles toward the left. The proprioceptive positioning was absent in the right thoracic and pelvic limbs. The remainder of the neurological examination was unremarkable, and a neurolocalization of left forebrain was determined.

Routine hematology was within normal limits, while serum biochemistry results included a moderately increased alkaline phosphatase activity (731 U/L; RI: 23-212 U/L). Noninvasive blood pressure (NIBP) assessment using Doppler identified a systolic pressure of 200–210 mmHg. An echocardiogram demonstrated mitral regurgitation consistent with presumed preclinical degenerative mitral valve disease. Urine-specific gravity was 1.020. Urine chemistry dipstick analysis was consistent with proteinuria (3+; RI: negative to trace), and a urine protein:creatinine (UPC) ratio was elevated (3.97; RI: 0-1). Urine sediment examination was normal, and urine culture was negative. Prothrombin time (PT), activated partial thromboplastin time (aPTT), fibrinogen, and D-dimer were all within normal limits. A shortened reaction time (*R*) and split point (SP) were seen on TEG analysis performed on citrated plasma (Table 1).

**Table 1:** Hemostatic parameters for ischemic stroke patients.

	**Case 1^**a**^**	**Case 2^**a**^**	**Case 3^**a**^**	**Case 4^**b**^**	**Case 5^**b**^**	**Reference interval**
Platelets (10^9^/L)	471	317	390	183^*^	288	200–500
PT (s)	5.7	8.6	6.8	7.5	6.8	<9.0
aPTT (s)	12.4	9.8	11.8	10.0	9.9	<12.5
Fibrinogen (g/L)	2.46	2.86	1.72	5.14^*^	1.67	1–4
D-dimer (mg/L)	<0.1	0.3	0.1	0.1	0.2	<0.5
**TEG parameters**						
*R* (min)	2.1^*^	2.6^*^	2.1^*^	5.4	2.7^*^	2.97–4.75^c^; 2.8–8.7^d^
*K* (min)	–	–	–	1.5^*^	1.8^*^	2.3–7.7^d^
SP (min)	1.9^*^	2.1^*^	1.8^*^	–	–	2.51–3.81^c^
α (°)	57.9	51.2	58.3	70^*^	65.5^*^	37.6–58.4^c^; 27.5–58.7^d^
MA (mm)	17.4	17.1	16.84	69.3^*^	62.1^*^	12.18–17.82^c^; 39–59^d^
*G* (dyn/cm^2^)	–	–	–	11268^*^	8182^*^	3200–7200^d^

*aPTT activated partial thromboplastin time; G global clot strength; K clotting time; MA maximum amplitude; PT prothrombin time; R reaction time; SP split point; TEG thromboelastography; α angle*.

a *TEG performed on citrated plasma*.

b *TEG performed on citrated whole blood*.

c *Reference values for citrated plasma (22)*.

d *Reference values for citrated whole blood (23)*.

* *Indicates values outside the reference range*.

**Figure 1:**
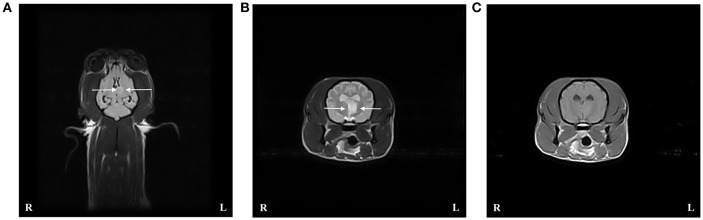
Case 1 T2-weighted (T2W) FLAIR dorsal image of the brain **(A)**, and T2W **(B)** and T1-weighted (T1W) post contrast **(C)** transverse images at the level of the thalamus revealed an intra-axial well demarcated lesion to the left of the midline consistent with a dorsal extensive thalamic infract. The lesion (indicated by arrows) is hyperintense compared to normal gray matter on T2W sequences and hypo- to isointense on T1W sequences.

An MRI scan of the brain (VetMR Grande 0.35T, Esaote, Genova, Italy) was performed under general anesthesia and included T2-weighted (T2W) transverse images, fluid attenuated inversion recovery (FLAIR) dorsal images, and short-TI inversion recovery transverse images. Sagittal, transverse and dorsal T1-weighted (T1W) images were acquired before and after intravenous (IV) administration of gadolinium contrast (0.1 mmol/kg, gadolinium demeglumine). An intra-axial, focal, well-defined and sharply demarcated lesion was identified within the left thalamus (Figure 1). The lesion was hypo- to isointense compared to normal gray matter on T1W images and hyperintense on T2W and FLAIR sequences. The lesion did not show enhancement after gadolinium administration, and there was no evidence of mass effect. These findings were suggestive of an extensive dorsal thalamic infarct.

Further investigations were performed to evaluate for evidence of predisposing causes for cerebrovascular disease and hypertension. Bilateral adrenomegaly was seen on abdominal ultrasound and a low-dose dexamethasone suppression test [basal cortisol: 204 nmol/L (RI: 27.5–125 nmol/L), 4 h post suppression: 34 nmol/L (RI: <40 nmol/L), 8 h post suppression: 168 nmol/L (RI: <40 nmol/L)] was consistent with pituitary dependent hyperadrenocorticism. Thoracic radiographs were unremarkable.

Treatment consisted of amlodipine (1 mg/kg PO q12 h; Istin 5 mg tablets, Pfizer Limited, Kent, UK), enalapril (0.5 mg/kg PO q24h; Enacard 2.5 mg tablets, Merial Animal Health, Harlow Essex, UK) and trilostane (5 mg/kg PO q24h; Vetoryl 30 mg tablets, Dechra, Shrewsbury, UK). The dog was hospitalized for seven days and during this time showed a gradual neurological improvement; the systolic blood pressure had stabilized around 170 mmHg at discharge. On reassessment 10 days after discharge, the dog was only showing mild proprioceptive deficits on the right side. The dog was lost to follow-up thereafter.

### Case 2

A 10-year, 10-month-old male neutered Shih Tzu presented for further investigation of a peracute onset of lethargy and left-sided circling. The general physical examination was normal. On neurological examination, the dog was circling to the left, had decreased proprioceptive positioning of the right thoracic and pelvic limbs and a decreased menace response on the right side. The remainder of the neurological examination was unremarkable, and the findings were considered consistent with a left forebrain neurolocalization.

Routine hematology and serum biochemistry was normal, and serum thyroxin and thyroid-stimulating hormone (TSH) concentrations were within normal limits. Urine-specific gravity was 1.018, urine chemistry dipstick analysis and sediment examination, UPC ratio and urine culture were all unremarkable. Assessment of NIBP using Doppler identified a systolic pressure of 170 mmHg. Fibrinogen, PT, aPTT, and D-dimer were all within normal limits. A shortened *R* time and SP were seen on TEG analysis on citrated plasma (Table 1).

An MRI scan of the brain using the same protocol and scanner as in case 1 was performed under general anesthesia. There was an extensive intra-axial, focal, well-demarcated lesion affecting the cerebral gray matter extending from the left frontal lobe to the rostral aspects of the left temporal lobe. The lesion was T2W and FLAIR hyperintense compared to normal gray matter and T1W hypointense. There was evidence of mild mass effect and there was no contrast enhancement. The findings were consistent with an ischemic stroke affecting the vascular territory of the left middle cerebral artery. CSF collected from the cerebellomedullary cistern was unremarkable. Abdominal ultrasound and thoracic radiographs were performed to evaluate for predisposing causes of the cerebrovascular event. No abnormalities were detected and accordingly no underlying cause for the ischemic stroke could be identified.

The dog improved with time and was discharged from hospital 72 h after admission. The dog made a near to full recovery within 14 days, and was neurologically normal at re-assessment five months later.

### Case 3

A seven-year, five-month-old male neutered Shih Tzu presented for further investigation of a peracute onset of a head tilt and loss of balance. The general physical examination was normal. On neurological examination, the dog was lethargic with a moderate left head tilt. The dog was non-ambulatory due to severe ataxia affecting all four limbs; with support the dog was dysmetric with hypermetria affecting the right thoracic and pelvic limbs. The remainder of the neurological examination was unremarkable, and a neurolocalization of right cerebellum was made, suspecting paradoxical vestibular disease.

Routine hematology, serum biochemistry, and standard urinalysis were normal. Serum thyroxin, TSH and an ACTH stimulation test were also within normal limits. Assessment of NIBP using Doppler identified a systolic pressure of 160–165 mmHg. Fibrinogen, PT, aPTT, and D-dimer were all within normal limits. A shortened *R* time and SP were seen on TEG analyses performed on citrated plasma (Table 1).

**Figure 2:**
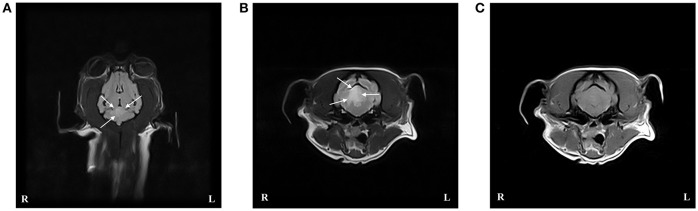
Case 3 T2-weighted (T2W) FLAIR dorsal image of the brain **(A)**, and T2W **(B)**, and T1-weighted (T1W) post contrast **(C)** transverse images at the level of the caudal cerebellar peduncles revealed an intra-axial well demarcated lesion to the right of the midline consistent with an ischemic stroke affecting the right rostral cerebellar artery. The lesion (indicated by arrows) is hyperintense compared to normal gray matter on T2W sequences and hypo- to isointense on T1W sequences.

An MRI scan of the brain using the same protocol and scanner as in case 1 was performed under general anesthesia. An intra-axial, focal, well-defined and sharply demarcated lesion was identified within the right cerebellar hemisphere (Figure 2). The lesion was hypointense compared to normal gray matter on T1W images, hyperintense on T2W, and FLAIR sequences and mainly affected the gray matter. The lesion did not show enhancement after gadolinium administration, and there was no evidence of mass effect. These findings were consistent with an ischemic stroke affecting the territory of the right rostral cerebellar artery. Supracollicular fluid accumulation was also evident, a finding that was considered to be incidental. CSF collected from the cerebellomedullary cistern was unremarkable. No underlying cause for the ischemic stroke could be identified.

The dog improved with time and was discharged from hospital after five days. On re-examination 14 days after discharge the dog was only showing mild hypermetria of the right thoracic limb, he was lost to follow-up thereafter.

### Case 4

A six-year, two-month-old female cavalier King Charles spaniel presented with a history of a peracute onset of lethargy and circling to the right. The dog presented to the hospital seven days after the onset of clinical signs, and had already improved clinically, according to the owner. The general physical examination was normal. On neurological examination, the dog showed delayed proprioceptive positioning on the left thoracic and pelvic limbs and an inconsistent menace response on the left side. Circling was not detected on presentation. The remainder of the neurological examination was normal, and the findings were considered consistent with a right forebrain neurolocalization.

**Figure 3:**
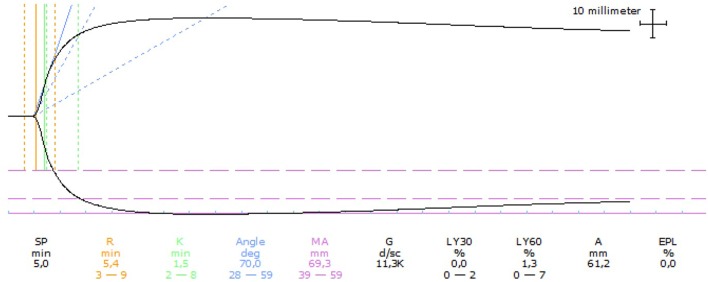
Thromboelastographic (TEG) tracing for case 4 performed on citrated whole blood. The TEG was considered hypercoagulable based on a normal reaction time (R), shortened clotting time (K), increased angle and maximum amplitude (MA), and increased clot strength (G). Dotted lines represent reference intervals and solid lines represent the test result.

Mild thrombocytopenia with the presence of macrothrombocytes was seen on routine hematology (Table 1). Serum biochemistry and standard urinalysis were unremarkable. Serum thyroxin and TSH were also within normal limits. Assessment of NIBP using oscillometry identified a systolic pressure of 170 mmHg. D-dimer, PT and aPTT were within normal limits whereas the fibrinogen level was mildly increased (Table 1). TEG analysis performed on citrated whole blood was considered hypercoagulable with a normal *R* time, shortened clotting time (*K*) time, and increased maximum amplitude (MA), angle (α), and global clot strength (*G* value) (Table 1). The TEG tracing is shown in Figure 3.

An MRI scan of the brain (VetMR 0.2T, Esaote, Genova, Italy) was performed under general anesthesia and included T2W sagittal and transverse images and FLAIR transverse images. Sagittal and transverse T1W images were acquired before and after IV administration of gadolinium contrast (0.1 mmol/kg, gadolinium dimeglumine). In the right frontal and rostral aspects of the temporal lobe there was an intra-axial, focal, well-defined, and sharply demarcated lesion. The lesion was hyperintense on T2W and FLAIR sequences and hypointense on T1W images compared to normal gray matter. The changes were particularly evident within the cortical gray matter. The lesion was associated with mild mass effect and there was no contrast enhancement. These findings were suggestive of an ischemic stroke affecting the territory of the right middle cerebral artery. Changes consistent with Chiari-like malformation and a left-sided otitis media with effusion were also seen; both were considered incidental findings. Further investigations to evaluate for a predisposing cause for cerebrovascular disease was not undertaken based on the owner's decision.

The dog improved with time, and was considered neurologically normal at re-examination 49 days after discharge, with no recurrence of neurological deficits.

### Case 5

A six-year, 11-month-old male Weimaraner presented with a five-day history of a peracute onset of a right-sided head tilt and loss of balance. The signs were improving on presentation. The general physical examination was normal. On neurological examination, the dog had an alert mentation, a mild right-sided head tilt, cerebellovestibular ataxia, and a broad-based stance affecting all limbs. There was occasional falling to the right with delayed and dysmetric hopping affecting all limbs. The remainder of the neurological examination was unremarkable, and a neurolocalization of right cerebellum was made.

Routine hematology, serum biochemistry, and standard urinalysis were unremarkable. Serum thyroxin and TSH were also within normal limits. Assessment of NIBP using oscillometry identified a systolic pressure of 170–175 mmHg. Fibrinogen, PT, aPTT, and D-dimer were all within normal limits. TEG analysis on citrated whole blood was considered hypercoagulable with a shortened *R* and *K* time, and an increased MA, α, and *G* value (Table 1).

An MRI scan of the brain using the same protocol and scanner as in case 4 was performed under general anesthesia. There was an intra-axial, focal and sharply demarcated wedge-shaped lesion within the right paravermis and rostrodorsal aspect of the cerebellar cortex. The lesion was hyperintense on T2W and FLAIR images compared to normal gray matter, and T1W hypointense. There was no mass effect or evidence of enhancement after contrast medium administration. These findings were most consistent with an ischemic stroke affecting the territory of the right rostral cerebellar artery. CSF collected from the cerebellomedullary cistern was normal. No predisposing cause for the ischemic stroke could be identified in this case.

The dog improved with time and was considered neurologically normal on re-assessment two months after presentation.

## Discussion

In this report we describe five dogs with ischemic stroke, all with normal D-dimer concentrations, suggesting that D-dimer is of little value as a negative predictor for arterial thromboembolic disease in the central nervous system, as opposed to other thromboembolic diseases in dogs (13, 14). In humans, a normal D-dimer has been shown to be a valuable negative predictor to rule out intravascular thrombotic processes, in particular in cases of venous thromboembolic disease such as central venous thrombosis and deep venous thrombosis (4, 12, 24, 25). D-dimer has also been shown to be significantly elevated in human ischemic stroke compared to healthy control populations (5–11), however, a normal D-dimer cannot rule out a diagnosis of ischemic stroke in people (4). In dogs, cerebral venous occlusion is reported but is thought to be a rare cause of ischemia due to abundant anastomoses (26, 27). In the present cases, normal D-dimer levels may be explained by arterial rather than venous occlusions. Normal D-dimer values have also previously been described in eight dogs diagnosed with ischemic stroke; detailed results were, however, not reported (20, 21).

In the present study, the D-dimer levels in cases 4 and 5 were measured seven and five days after the onset of clinical signs, respectively, and it cannot be excluded that the late sampling influenced the measured concentrations. However, in human patients following ischemic stroke, D-dimer levels gradually increase peaking around two weeks after onset of clinical signs, remain high for several weeks, and then gradually decrease (4).

Two of the dogs in this case series were characterized as hypercoagulable based on shortened *R* and *K* times, and increased α and MA. The *G* value in these two dogs was >7,200 dyn/cm^2^ also indicating an increased clot strength (23). In the remaining three cases, TEG analysis was performed on citrated plasma which may lack important cellular factors. Even though it is more difficult to apply these results in a clinical setting, all three dogs had a shortened *R* time and SP, compared to a previously established reference range on plasma TEG, indicating a hypercoagulable state also in these dogs.

TEG has also been investigated in human ischemic stroke, where the analyses suggest the existence of a hypercoagulable state in 29–41% of the patients (18, 28). Acute ischemic stroke patients with hypercoagulable TEG had shorter *R* and *K* times and a greater α indicating a faster clotting. Variable clot strength was detected in these patients, with 24.5% having an increased clot strength indicated by an elevated *G* value (18). In canine stroke, the use of TEG has only been reported in four greyhounds diagnosed with ischemic stroke (20). The analysis was reported to be normal in these dogs compared to previously reported reference values (20, 29). Greyhounds are, however, reported to have slower clotting kinetics and weaker clot strengths compared to non-greyhound dogs (29). The TEG results from greyhounds with ischemic stroke may therefore not be representative for non-greyhound dogs with the same diagnosis.

A hypercoagulable state has previously been suspected in four dogs diagnosed with ischemic stroke based on routine hemostatic assays and comorbidities (19). Routine hemostatic assays, however, have poor sensitivity for the detection of hypercoagulability, whereas TEG has been evaluated for its capacity to provide early, sensitive detection of a hypercoagulable state (15). Even though the results in the present study are based on a small sample size, the demonstration of a possible hypercoagulable state as suggested by the TEG results is an interesting finding, which should be explored further to help reveal predisposing hypercoagulable conditions in dogs with ischemic stroke. It is, however, difficult to know if the TEG results predated the stroke or if they were a result thereof.

Hypercoagulable conditions can cause thromboembolic disease, and predisposing causes of hypercoagulable states such as hyperadrenocorticism, protein-losing nephropathy, splenic hemangiosarcoma, and bacterial endocarditis have been described in dogs with ischemic stroke (19, 30–32). In this case series, only one dog was diagnosed with a comorbidity (pituitary-dependent hyperadrenocorticism) that could explain a hypercoagulable state. The dogs were, however, not evaluated for primary hypercoagulable states.

The diagnosis of ischemic stroke in the current cases was based on the peracute presentation of focal non-progressive neurological signs and imaging characteristics on conventional MRI sequences. Diffusion-weighted and gradient echo imaging were however not performed due to the low field strength magnets used for these cases. This is a limitation as additional hemorrhagic lesions cannot completely be excluded.

In conclusion, a normal D-dimer does not seem an appropriate negative predictor for canine ischemic stroke and should not replace either clinical or radiological evaluation. The application of TEG to canine ischemic stroke patients may contribute with important information on the global hemostatic conditions of these animals and may eventually be used to guide and monitor thrombolytic therapy.

## Consent for Publication

Written informed consent for publication were collected from the owner of the patient in all cases.

## Data Availability

The raw data supporting the conclusions of this manuscript will be made available by the authors, without undue reservation, to any qualified researcher.

## Ethics Statement

This study was carried out in accordance with and approved by the Local Administrative and Ethics Committee, Department of Veterinary Clinical Sciences, University of Copenhagen.

## Author Contributions

BK interpreted the data, contributed to article concept, and drafted the manuscript. LM made a substantial contribution to the acquisition of data. BW and AK performed the hemostatic assessments and interpreted data. UW helped with data acquisition and performed the MRI assessments. GS, MB, and HG contributed to the article concept and acquisition of data. All authors contributed to the manuscript revision and have read and approved the submitted manuscript.

### Conflict of Interest Statement

BW is employed by company Novo Nordisk. The remaining authors declare that the research was conducted in the absence of any commercial or financial relationships that could be construed as a potential conflict of interest.

## References

[1] World Health Organization WHO STEPS Stroke Manual: The WHO STEPwise Approach to Stroke Surveillance (2006).

[2] Garosi L McConnell JF Platt SR Barone G Baron JC de Lahunta A. Clinical and topographic magnetic resonance characteristics of suspected brain infarction in 40 dogs. J Vet Intern Med. (2006) 20:311–21. 10.1111/j.1939-1676.2006.tb02862.x 16594588

[3] Gonçalves R Carrera I Garosi L Smith PM McConnell F Penderis J. Clinical and topographic magnetic resonance imaging characteristics of suspected thalamic infarcts in 16 dogs. Vet J. (2011) 188:39–43. 10.1016/j.tvjl.2010.03.024 20456988

[4] Haapaniemi E Tatlisumak T. Is D-dimer helpful in evaluating stroke patients? A systematic review. Acta Neurol Scand. (2009) 119:141–50. 10.1111/j.1600-0404.2008.01081.x 18705677

[5] Ono N Koyama T Suehiro A Oku K Fujikake K Kakishita E. Clinical significance of new coagulation and fibrinolytic markers in ischemic stroke patients. Stroke. (1991) 22:1369–73. 10.1161/01.STR.22.11.1369 1836283

[6] Altès A Mbellán MT Mateo J Avila A Martí-Vilalta JL Fontcuberta J. Hemostatic disturbances in acute ischemic stroke: a study of 86 patients. Acta Haematol. (1995) 94:10–5. 10.1159/000203964 7653207

[7] Takano K Yamaguchi T Kato H Omae T. Activation of coagulation in acute cardioembolic stroke. Stroke. (1991) 22:12–6. 10.1161/01.STR.22.1.12 1987667

[8] Ince B Bayram C Harmanci H Ulutin T. Hemostatic markers in ischemic stroke of undetermined etiology. Thromb Res. (1999) 96:169–74. 10.1016/S0049-3848(99)00097-3 10588458

[9] Kataoka S Hirose G Hori A Shirakawa T Saigan T. Activation of thrombosis and fibrinolysis following brain infarction. J Neurol Sci. (2000) 181:82–8. 10.1016/S0022-510X(00)00435-4 11099716

[10] Tombol T Atbas C Anlar O Hemostatic markers and platelet aggregation factors as predictive markers for type of stroke and neurological disability following cerebral infarction. J Neurol Sci. (2005) 12:429–34. 10.1016/j.jocn.2004.06.013 15925775

[11] Li J Gu C Li D Chen L Lu Z Zhu L. Effects of serum N-terminal pro B-type natriuretic peptide and D-dimer levels on patients with acute ischemic stroke. Pak J Med Sci. (2018) 34:994–8. 10.12669/pjms.344.15432 30190768 PMC6115578

[12] Chapman C Akhtar N Campbell S Miles K O'Connor J Mitchell VE. The use of D-dimer assay by enzyme immunoassay and latex agglutination techniques in the diagnosis of deep vein thrombosis. Clin Lab Haematol. (1990) 12:37–42. 10.1111/j.1365-2257.1990.tb01108.x 2188775

[13] Nelson OL Andreasen C. The utility of plasma D-dimer to identify thromboembolic disease in dogs. J Vet Intern Med. (2003) 17:830–4. 10.1111/j.1939-1676.2003.tb02522.x 14658720

[14] Nelson OL. Use of the D-dimer assay for diagnosing thromboembolic disease in the dog. J Am Anim Hosp Assoc. (2005) 41:145–9. 10.5326/0410145 15870247

[15] Kol A Borjesson DL Application of thromboelastography/thromboelastometry to veterinary medicine. Vet Clin Pathol. (2010) 39:405–16. 10.1111/j.1939-165X.2010.00263.x 20969608

[16] Wiinberg B Jensen AL Johansson PI Rozanski E Tranholm M Kristensen AT. Thromboelastographic evaluation of hemostatic function in dogs with disseminated intravascular coagulation. J Vet Intern Med. (2008) 22:357–65. 10.1111/j.1939-1676.2008.0058.x 18346141

[17] Wiinberg B Jensen AL Rozanski E Johansson PL Kjelgaard-Hansen M Tranholm M. Tissue factor activated thromboelastography correlates to clinical signs of bleeding in dogs. Vet J. (2009) 179:121–9. 10.1016/j.tvjl.2007.08.022 17920966

[18] Elliott A Wetzel J Roper T Pivalizza E McCarthy J Wallace C. Thromboelastography in patients with acute ischemic stroke. Int J Stroke. (2015) 10:194–201. 10.1111/j.1747-4949.2012.00919.x 23017088 PMC3535538

[19] Garosi L McConnell JF Platt SR Barone G Baron JC de Lahunta A. Results of diagnostic investigations and long-term outcome of 33 dogs with brain infarction (2000-2004). J Vet Intern Med. (2005) 19:725–31. 10.1111/j.1939-1676.2005.tb02752.x 16231718

[20] Kent M Glass EN Haley AC March P Rozanski EA Galban EM. Ischemic stroke in Greyhounds: 21 cases (2007-2013). J Am Vet Med Assoc. (2014) 245:113–7. 10.2460/javma.245.1.113 24941395

[21] Rossmeisl JH Jr Rohleder JJ Pickett JP Duncan R Herring IP. Presumed and confirmed striatocapsular brain infarctions in six dogs. Vet Ophthalmol. (2007) 10:23–36. 10.1111/j.1463-5224.2007.00487.x 17204125

[22] Wiinberg B Jensen AL Kjelgaard-Hansen M Rojkjaer R Johansson PI Gade LP. Study on biological variation of haemostatic parameters in clinically healthy dogs. Vet J. (2007) 174:62–8. 10.1016/j.tvjl.2006.05.003 16815052

[23] Wiinberg B Jensen AL Rojkjaer R Johansson P Kjelgaard-Hansen M Kristensen AT. Validation of human recombinant tissue factor-activated thromboelastography on citrated whole blood from clinically healthy dogs. Vet Clin Pathol. (2005) 34:389–93. 10.1111/j.1939-165X.2005.tb00066.x 16270265

[24] Saposnik G Barinagarrementeria F Brown RD Bushnell CD Cucchiara B Cushman M. Diagnosis and management of cerebral venous thrombosis: a statement for healthcare professionals from the American Heart Association/American Stroke Association. Stroke. (2011) 42:1158–92. 10.1161/STR.0b013e31820a8364 21293023

[25] Kosinski CM Mull M Schwarz M Koch B Biniek R Schläfer J. Do normal D-dimer levels reliably exclude cerebral sinus thrombosis? Stroke. (2004) 35:2820–5. 10.1161/01.STR.0000147045.71923.18 15514174

[26] Swayne DE Tyler DE Batker J. Cerebral infarction with associated venous thrombosis in a dog. Vet Pathol. (1988) 25:317–20. 10.1177/030098588802500413 3407103

[27] Boudreau CE. An update on cerebrovascular disease in dogs and cats. Vet Clin Small Anim. (2018) 48:45–62. 10.1016/j.cvsm.2017.08.009 29056397

[28] Ettinger MG. Thromboelastographic studies in cerebral infarction. Stroke. (1974) 5:350–4. 10.1161/01.STR.5.3.350 4836538

[29] Vilar P Couto CG Westendorf N Iazbik C Charske J Marín L Thromboelastographic tracings in retired racing greyhounds and in non-greyhound dogs. J Vet Intern Med. (2008) 25:374–9. 10.1111/j.1939-1676.2008.0061.x 18346139

[30] Hackner SG Schaer BD Thrombotic disorders. In: Weiss DJ Wardrop KJ editors. Schalm's Veterinary Hematology, 6th Edn. Ames, IA: Wiley-Blackwell (2010). p. 668–78.

[31] Sykes JE Kittleson MD Chomel BB Macdonald KA Pesavento PA Clinicopathological findings and outcome in dogs with infective endocarditis: 71 cases (1992-2005). J Am Vet Med Assoc. (2006) 228:1735–47. 10.2460/javma.228.11.1735 16740075

[32] Cook LB Coates JR Dewey CW Gordon S Miller MW Bahr A. Vascular encephalopathy associated with bacterial endocarditis in four dogs. J Am Anim Hosp Assoc. (2005) 41:252–8. 10.5326/0410252 15995163

